# The value of sex hormones and sex hormone-binding globulin in metabolic dysfunction-associated fatty liver disease among boys with obesity

**DOI:** 10.3389/fendo.2025.1446049

**Published:** 2025-02-06

**Authors:** Ying Wang, Shuyi Yang, Suming Zhang, Ye Yang, Siqing Li, Meiyu Zhang, Xiaona Li, Hua Bai, Peiliang Luo, Yingdi Yuan

**Affiliations:** ^1^ Pediatric Endocrinology Department, The First People’s Hospital of Lianyungang, The Affiliated Lianyungang Hospital of Xuzhou Medical University, Lianyungang, China; ^2^ Department of Pediatrics, Lianyungang Municipal Oriental Hospital, Lianyungang, China; ^3^ Pediatric Endocrinology Department, The First People’s Hospital of Lianyungang, Lianyungang Clinical College of Nanjing Medical University, Lianyungang, China

**Keywords:** childhood, obesity, metabolic dysfunction-associated fatty liver disease (MAFLD), non-alcoholic fatty liver disease (NAFLD), sex hormone-binding globulin (SHBG), testosterone

## Abstract

**Objective:**

This study aims to investigate the relationship between metabolic dysfunction-associated fatty liver disease (MAFLD) and sex hormones and sex hormone-binding globulin (SHBG) in boys with obesity.

**Methods:**

Retrospective analysis of metabolic indicators and sex hormone levels in boys with obesity who sought medical attention at the First People’s Hospital of Lianyungang City from January 2020 to December 2023. Based on abdominal ultrasound results, they were categorized into a simple obesity group and MAFLD group, and differences between the two groups were compared. Utilizing logistic regression analysis to explore the risk factors for developing MAFLD, and through the construction of Receiver Operating Characteristic (ROC) curves, conducting a preliminary assessment of the diagnostic value for MAFLD.

**Results:**

A total of 155 male children with obesity were included in the study, mean age of 11.07 ± 1.53 years. Children in the MAFLD group had higher levels of height[(159.49 ± 12.73)cm vs.(155.55 ± 10.50)cm], weight[(82.32 ± 18.75)kg vs.(68.28 ± 15.00)kg], BMI[(32.08 ± 4.49)kg/m^2^ vs.(27.85 ± 4.21)kg/m^2^],fasting insulin[33.42(24.07,43.93)uIU/ml vs.23.91(15.72,31.52)uIU/ml],HOMA-IR[7.27(5.26,10.71) vs.4.87(3.27,6.86)],fastingC-peptide[1409.00(1175.00,1668.00)pmol/L vs.1020.00(849.05,1303.00)pmol/L], WBC[(7.85 ± 1.80)×10^9^/L vs.(7.15 ± 1.42)×10^9^/L], HbA1c[5.40(5.30,5.70)% vs.(5.30(5.20,5.60)%],ALT[48.00(27.00,80.00)U/L vs.19.00(15.00,26.50)U/L], and AST[31.00(24.00,60.00)U/L vs.21.00(18.50, 26.00)U/L] compared to the simple obesity group (P<0.05). Children in the MAFLD group had lower levels of HDL[(1.05 ± 0.21)mmol/L vs.(1.16 ± 0.26)mmol/L], testosterone [42.41(30.33,143.28)ng/dl vs.125.41(23.41,221.57)ng/dl], and SHBG[13.20(9.10,17.30)nmol/l vs.19.60(13.50,29.85)nmol/l] compared to the simple obesity group (P<0.05). Logistic regression showed that BMI, testosterone, and SHBG were independent risk factors for MAFLD in boys, and ROC curve analysis indicated their potential value in the early diagnosis of MAFLD.

**Conclusion:**

BMI, testosterone, and SHBG are independent risk factors for the occurrence of MAFLD in boys with obesity. To control the occurrence of MAFLD, it is essential to address the root cause of the high growth rate of obesity. The roles of testosterone and SHBG in MAFLD merit further research.

## 1 Introduction

As is well known, both obesity and non-alcoholic fatty liver disease (NAFLD) are significant global public health issues (1, 2), and NAFLD is closely associated with obesity. With increasingly comprehensive research, it has been found that NAFLD is a manifestation of systemic metabolic dysfunction. There are differences in histology and diagnosis between adult and pediatric NAFLD. Children are generally not affected by alcohol in their daily lives, indicating a drawback in the previous nomenclature of NAFLD. Consequently, experts in fatty liver and pediatric specialists have proposed a new name and diagnostic criteria for metabolic (dysfunction)-associated fatty liver disease (MAFLD) (3, 4). NAFLD/MAFLD has become a major cause of chronic liver disease worldwide, and in severe cases, it can lead to liver fibrosis, cirrhosis, liver cell cancer, and a series of liver diseases. It is also a risk factor for metabolic diseases such as cardiovascular disease, extrahepatic cancer, and type 2 diabetes (5–7). The liver is the center of many physiological processes and is also a target for sex hormones such as testosterone and estrogen. These hormones participate in liver metabolism and immune regulation through various pathways, thereby influencing the occurrence of liver diseases and related diseases (8, 9). The relationship between sex hormones and SHBG with adult NAFLD/MAFLD has become a research hotspot (10–12), but there is limited research in the pediatric population. This article aims to explore the relationship between sex hormones and SHBG in MAFLD in boys, attempting to identify new intervention targets for MAFLD.

## 2 Materials and methods

### 2.1 Clinical subject

Children diagnosed with obesity based on body mass index (BMI) measurements conducted by specialized endocrinologists in the Pediatric Endocrinology Department of the First People’s Hospital of Lianyungang City from January 2020 to December 2023 were selected as the subjects for this study. The BMI of all participants met the diagnostic criteria for childhood obesity (13). Participants with the following conditions were excluded: 1. Partial research data missing. 2. Long-term use of corticosteroids or other medications affecting hormonal levels. 3. History of genetic or endocrine disorders. A total of 155 male children with obesity were included in the study, with an age range of 8-14 years and a mean age of 11.07 ± 1.53 years. Based on fasting ultrasound results, participants were divided into the MAFLD group (86 cases) if their ultrasound met the criteria for fatty live (14), otherwise, they were included in the simple obesity group (69 cases).

### 2.2 Clinical data and laboratory examination

Shenzhen Mindray 7500 fully automatic hematology analyzer was used to determine blood routine and CRP, Beckman Coulter fully automatic biochemical analyzer was used to measure blood sugar, blood lipids, and liver and kidney function, while Beckman Coulter chemiluminescence analyzer was used to measure hormones, thyroid function, etc. VARIANT II TURBO hemoglobin A1c analyzer was used to measure HbA1c. Ultrasound examinations were performed using Philips EQ7, GE e20, or GE e10 ultrasound machines. Information on examinations and test results of the patients was extracted and analyzed through the medical record system, including gender, age, height, weight, BMI, etc.

### 2.3 Statistical analysis

The data were analyzed using SPSS 27.0 statistical software. For normally distributed continuous data, they were expressed as ( $\bar{\chi }\pm s$ ). The independent samples t-test was used for comparisons between two groups. For non-normally distributed continuous data, the median and interquartile range were represented as M[P25, P75], and the Mann-Whitney U test was employed for comparisons between two groups. Binary Logistic regression models were used to calculate the risk factors for childhood MAFLD, and Receiver Operating Characteristic (ROC) curves were plotted to evaluate the predictive value of different factors for MAFLD. A *P* value < 0.05 was considered statistically significant.

## 3 Results

The difference in age between the two groups of children was not statistically significant (*P* > 0.05). Children in the MAFLD group had higher values for height, weight, and BMI compared to the simple obesity group, and the differences between the groups were statistically significant (*P* < 0.05) (**Table 1**). Children in the MAFLD group had higher levels of fasting insulin (FINS), HOMA-IR, fasting C-peptide, leukocyte (WBC), HbA1c, alanine aminotransferase (ALT), and aspartate aminotransferase (AST) compared to the simple obesity group. However, levels of high-density lipoprotein cholesterol (HDL), testosterone, and SHBG were lower in the MAFLD group than in the simple obesity group. These differences were statistically significant (*P* < 0.05). There were no statistically significant differences (*P* > 0.05) between the two groups in terms of fasting blood sugar (FBG), C-reactive protein (CRP), total cholesterol (TC), triglycerides (TG),low-density lipoprotein cholesterol (LDL), Estradiol(E2), luteinizing hormone(LH), follicle stimulating hormone (FSH),prolactin (PRL), and progesterone (**Table 2**).

**Table 1:** Baseline demographics for the patients with MAFLD and controls.

Group	MAFLD group	Simple obesity group	t/ *x* ^2^	*P*
age (years)	11.24±1.45	10.86±1.61	1.530	0.128
height (cm)	159.49±12.73	155.55±10.50	2.063	0.041
weight (kg)	82.32±18.75	68.28±15.00	5.057	<0.001
BMI (kg/m2)	32.08±4.49	27.85±4.21	5.981	<0.001

BMI, body mass index.

**Table 2:** Clinical characteristics for the patients with MAFLD and controls.

Group	MAFLD group	Simple obesity group	t/Z	*P*
FINS (uIU/ml)	33.42 (24.07,43.93)	23.91 (15.72,31.52)	-4.596	<0.001
FBG (mmol/L)	4.86 (4.65,5.06)	4.79 (4.55,5.06)	-0.916	0.359
HOMA-IR	7.27 (5.26,10.71)	4.87 (3.27,6.86)	-4.807	<0.001
Fasting C-peptide (pmol/L)	1409.00 (1175.00,1668.00)	1020.00 (849.05,1303.00)	-5.359	<0.001
WBC (×10^9^/L)	7.85±1.80	7.15±1.42	2.633	0.009
CRP (mg/L)	2.94 (1.57,4.70)	2.10 (1.17,4.47)	-1.534	0.125
TC (mmol/L)	4.17 (3.66,4.87)	4.36 (3.89,4.90)	-0.861	0.389
TG (mmol/L)	1.35 (0.97,1.93)	1.24 (0.80,1.68)	-1.102	0.271
HDL (mmol/L)	1.05±0.21	1.16±0.26	-2.625	0.010
LDL (mmol/L)	2.62±0.68	2.62±0.65	0.022	0.982
HbA1c (%)	5.40 (5.30,5.70)	5.30 (5.20,5.60)	-1.971	0.049
ALT (U/L)	48.00 (27.00,80.00)	19.00 (15.00,26.50)	-6.560	<0.001
AST (U/L)	31.00 (24.00,60.00)	21.00 (18.50,26.00)	-5.601	<0.001
testosteron (ng/dl)	42.41 (30.33,143.28)	125.41 (23.41,221.57)	-1.979	0.048
E2 (pg/ml)	15.00 (15.00,21.00)	15.00 (15.00,21.69)	-0.403	0.687
LH (mIU/ml)	2.31 (0.38,3.79)	1.73 (0.29,3.60)	-1.106	0.269
FSH (mIU/ml)	3.99±2.64	3.85±2.44	0.331	0.741
PRL (ng/ml)	14.19±7.26	13.61±6.94	0.504	0.615
progesterone (ng/ml)	0.45 (0.26,0.84)	0.53 (0.36,0.70)	-0.767	0.443
SHBG (nmol/l)	13.20 (9.10,17.30)	19.60 (13.50,29.85)	-4.513	<0.001

FINS, fasting insulin; FBG, fasting blood sugar; WBC, leukocyte; CRP, C-reactive protein; TC, total cholesterol; TG, triglycerides; HDL, high-density lipoprotein cholesterol; LDL, low-density lipoprotein cholesterol; ALT, alanine aminotransferase; AST, aspartate aminotransferase; E2, Estradiol; LH, luteinizing hormone; FSH, follicle stimulating hormone; PRL, prolactin; SHBG, sex hormone-binding globulin.

A univariate analysis was conducted with the presence or absence of MAFLD as the dependent variable (using *P* < 0.05 as the inclusion criterion). Then, multiple-factor logistic regression analysis was performed, including BMI, fasting C-peptide, WBC, HDL, ALT, AST, testosterone, and SHBG. The results revealed that BMI, testosterone, and SHBG were independent risk factors for MAFLD in male children (**Table 3**).

**Table 3:** Logistic analysis of factors of MAFLD in children with obesity.

	*β*	*SE*	*Waldχ2*	*P*	*0R*	*95%CI*
BMI (kg/m2)	0.148	0.054	7.551	0.006	1.159	(1.043,1.288)
Fasting C-peptide (pmol/L)	0.000	0.001	0.029	0.865	1.000	(0.999,1.002)
WBC (×10^9^/L)	0.158	0.139	1.280	0.258	1.171	(0.891,1.538)
HDL (mmol/L)	-1.809	1.084	2.783	0.095	0.164	(0.020,1.372)
ALT (U/L)	0.028	0.019	2.196	0.138	1.029	(0.991,1.068)
AST (U/L)	-0.005	0.033	0.022	0.881	0.995	(0.933,1.061)
testosteron (ng/dl)	-0.007	0.003	7.573	0.006	0.993	(0.988,0.998)
SHBG (nmol/l)	-0.064	0.026	6.196	0.013	0.938	(0.892,0.987)
constant	-2.690	2.534	1.126	0.289	0.068	

BMI, body mass index; WBC, leukocyte; HDL, high-density lipoprotein cholesterol; ALT, alanine aminotransferase; AST, aspartate aminotransferase; SHBG, sex hormone-binding globulin.

**Figure 1:**
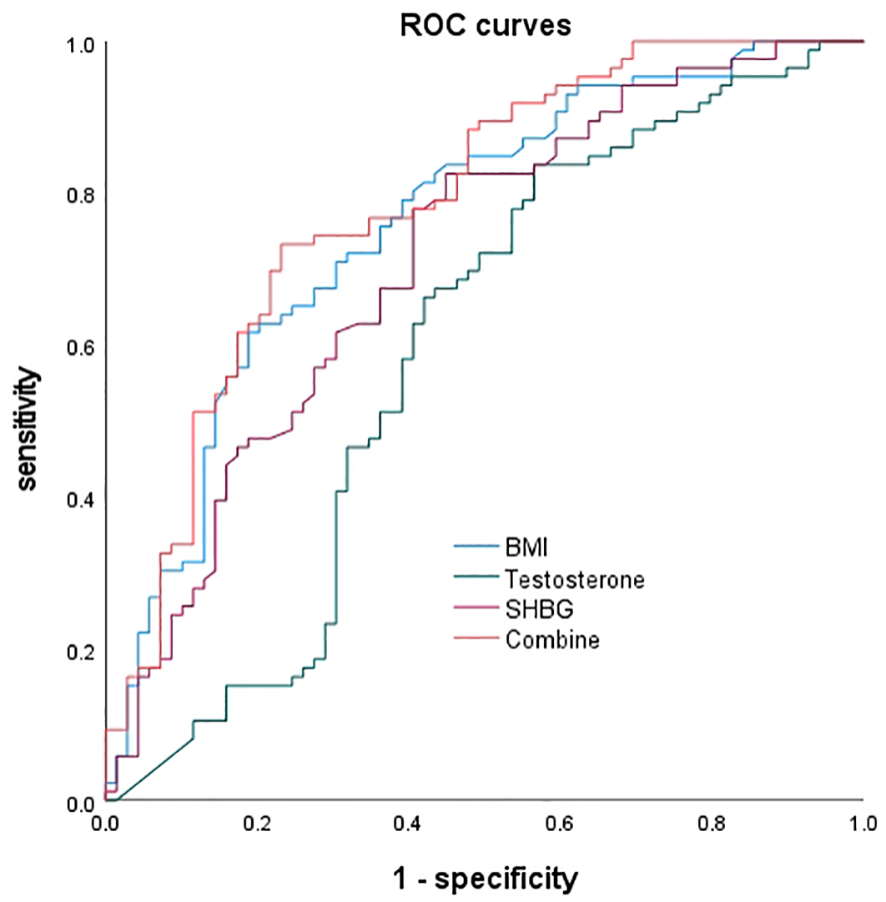
ROC curves.

The ROC curves were plotted, and for BMI alone predicting MAFLD, the area under the curve was 0.761, with a 95% CI of 0.685-0.838. The optimal cutoff value was 32.065, with a sensitivity of 61.6% and specificity of 81.2%. For testosterone alone predicting MAFLD, the area under the curve was 0.593, with a 95% CI of 0.498-0.687. The optimal cutoff value was 161.875, with a sensitivity of 83.7% and specificity of 43.5%. For SHBG alone predicting MAFLD, the area under the curve was 0.711, with a 95% CI of 0.629-0.794. The optimal cutoff value was 18.65, with a sensitivity of 82.6% and specificity of 55.1%. When the three factors were combined to predict NAFLD, the area under the curve was 0.787, with a 95% CI of 0.714-0.860, a sensitivity of 73.3%, and a specificity of 76.8% (**Figure 1**).

## 4 Discussion

Childhood and adolescent overweight/obesity are globally prevalent. According to the World Obesity Federation’s 2023 projection, the prevalence of overweight/obesity is expected to increase rapidly from 38% in 2020 to 51% in 2035 among the global population aged >5 years (15). Obesity rates are anticipated to rise from 14% in 2020 to 24% in 2035, with the number reaching nearly 2 billion (15).Childhood and adolescent overweight/obesity can pose risks to both short-term and long-term physical and mental health. If these risks are not effectively managed, they may progressively worsen and impact adult health, increasing the incidence of metabolic-related complications such as type 2 diabetes, cardiovascular diseases, MAFLD, and others (16, 17).The interrelationships among its complications are intricate, and in severe cases, they can lead to a reduction in lifespan and an increase in mortality rates. Further research is needed to delve into the specific pathogenic mechanisms and intervention measures.

In this study, we chose to analyze MAFLD one of the complications of obesity, and found that in the MAFLD group, male children had higher weight and BMI compared to the group with simple obesity, and these differences were statistically significant (*P* < 0.05), indicating that the prevalence is influenced by the severity of obesity. There exists a vicious cycle between the degree of obesity and insulin resistance. Obesity puts the body in a state of chronic inflammation, leading to insulin resistance, which in turn promotes fat accumulation and obesity (18–21). Various factors (including dietary habits, physical activity levels, sedentary behaviors, screen time, family dynamics, socio-economic status, and environmental factors) causing extensive fat accumulation result in increased weight and BMI in male children in the MAFLD group (22, 23). As obesity worsens, insulin resistance becomes more pronounced, leading to higher levels of FINS, fasting C-peptide, HOMA-IR, and WBC in the MAFLD group compared to the simple obesity group (24). The accumulation of a large amount of fat in the liver impairs its metabolic function, causing elevated levels of ALT and AST.

According to research reports, the prevalence of MAFLD among children ranges from 7.6% to 9.6%, while the prevalence among obese children is 34.2% (6).Research indicates that the prevalence of NAFLD is higher in boys than in girls (9% in boys compared to 6.3% in girls) and gradually increases with the rise in BMI (35.3% in obese boys compared to 21.8% in obese girls) (25).On the one hand, this may be attributed to a higher obesity rate in boys compared to girls. On the other hand, it is also suggested that sex hormones play a certain role in the occurrence and development of MAFLD. Some studies have confirmed that estrogen has a certain protective effect on the occurrence of NAFLD (26, 27).Testosterone, as a primary androgen, serves as a crucial regulator for reproductive organs and other tissues and is associated with cardiovascular diseases (28), diabetes (29), and metabolic syndrome (30).SHBG is a homodimeric glycoprotein produced by the liver. It exhibits strong binding affinity with testosterone and can regulate the activity of testosterone and other steroid hormones, assisting in their transportation into target tissues (31).In adults, researches has found a correlation between SHBG and the occurrence of NAFLD (11, 12, 32, 33) and polycystic ovary syndrome (34, 35). This study found a correlation between SHBG and MAFLD in male children, with its levels negatively correlated with BMI. After controlling for confounding factors, BMI, SHBG, and testosterone were identified as independent risk factors for MAFLD in male children, suggesting a predictive value for early diagnosis of MAFLD in children. Therefore, we hypothesize that appropriately increasing SHBG and testosterone levels may be beneficial for the control and intervention of MAFLD in male children. Consistent with our hypothesis, a recent Mendelian randomization analysis also indicated that high levels of SHBG can reduce the risk of NAFLD, suggesting SHBG levels as a useful biomarker (36). However, the specific mechanisms and intervention strategies between SHBG, testosterone, and MAFLD need further research.

This study also have some limitations. Firstly, it only included male children with obesity as the study subjects, with a relatively small sample size, which may not be representative of the entire pediatric population. Secondly, liver biopsy, a gold standard for diagnosing fat deposition, is not feasible in routine screening for children. Other novel non-invasive detection methods such as instantaneous elastography, magnetic resonance spectroscopy and cytokeratin 18 (CK-18) have not been promoted in clinical detection (37).Therefore, this study relied primarily on ultrasound results for the diagnosis of hepatic steatosis. Lastly, as this study was a retrospective analysis, some anthropometric data such as waist circumference, waist-to-hip ratio, and waist-to-height ratio were missing for some children during hospital admission examinations.

In conclusion, this study identified high BMI, low SHBG, and low testosterone as risk factors for MAFLD in male children. MAFLD during childhood can pose health risks in adulthood, emphasizing the critical importance of implementing effective screening, diagnosis, and intervention measures during childhood. Currently, there are no approved drugs for the treatment of pediatric MAFLD both domestically and internationally. The preferred interventions involve modifying dietary habits, adopting a healthier lifestyle, increasing physical activity, and implementing weight reduction measures to prevent obesity and subsequently reduce the incidence of MAFLD. This study suggests that, in addition to weight control, appropriately increasing testosterone and SHBG levels may serve as potential early intervention measures for MAFLD. Subsequent research will aim to expand the sample size or conduct *in vitro* studies.

## Data Availability

The raw data supporting the conclusions of this article will be made available by the authors, without undue reservation.

## References

[1] Shaunak M Byrne CD Davis N Afolabi P Faust SN Davies JH. Non-alcoholic fatty liver disease and childhood obesity. Arch Dis Child. (2021) 106:3–8. doi: 10.1136/archdischild-2019-318063 32409495

[2] Brecelj J Orel R. Non-alcoholic fatty liver disease in children. Medicina (Kaunas). (2021) 57:719. doi: 10.3390/medicina57070719 34357000 PMC8304730

[3] Eslam M Newsome PN Sarin SK Anstee QM Targher G Romero-Gomez M. A new definition for metabolic dysfunction-associated fatty liver disease: An international expert consensus statement. J Hepatol. (2020) 73:202–9. doi: 10.1016/j.jhep.2020.03.039 32278004

[4] Eslam M Alkhouri N Vajro P Baumann U Weiss R Socha P. Defining paediatric metabolic (dysfunction)-associated fatty liver disease: an international expert consensus statement. Lancet Gastroenterol Hepatol. (2021) 6:864–73. doi: 10.1016/S2468-1253(21)00183-7 34364544

[5] Han SK Baik SK Kim MY. Non-alcoholic fatty liver disease: Definition and subtypes. Clin Mol Hepatol. (2023) 29:S5–S16. doi: 10.3350/cmh.2022.0424 36577427 PMC10029964

[6] Le Garf S Nègre V Anty R Gual P. Metabolic fatty liver disease in children: A growing public health problem. Biomedicines. (2021) 9:1915. doi: 10.3390/biomedicines9121915 34944730 PMC8698722

[7] Badmus OO Hillhouse SA Anderson CD Hinds TD Stec DE. Molecular mechanisms of metabolic associated fatty liver disease (MAFLD): functional analysis of lipid metabolism pathways. Clin Sci (Lond). (2022) 136:1347–66. doi: 10.1042/CS20220572 PMC9508552 36148775

[8] Kasarinaite A Sinton M Saunders PTK Hay DC. The influence of sex hormones in liver function and disease. Cells. (2023) 12:1604. doi: 10.3390/cells12121604 37371074 PMC10296738

[9] Kur P Kolasa-Wołosiuk A Misiakiewicz-Has K Wiszniewska B. Sex hormone-dependent physiology and diseases of liver. Int J Environ Res Public Health. (2020) 17:2620. doi: 10.3390/ijerph17082620 32290381 PMC7216036

[10] Wang N Zhai H Zhu C Li Q Han B Chen Y. Combined association of vitamin D and sex hormone binding globulin with nonalcoholic fatty liver disease in men and postmenopausal women: A cross-sectional study. Med (Baltimore). (2016) 95:e2621. doi: 10.1097/MD.0000000000002621 PMC5291588 26825918

[11] Luo J Chen Q Shen T Wang X Fang W Wu X. Association of sex hormone-binding globulin with nonalcoholic fatty liver disease in Chinese adults. Nutr Metab (Lond). (2018) 15:79. doi: 10.1186/s12986-018-0313-8 30455723 PMC6225668

[12] Kim D Manikat R Cholankeril G Ahmed A. Endogenous sex hormones and nonalcoholic fatty liver disease in US adults. Liver Int. (2024) 44:460–71. doi: 10.1111/liv.15786 38010926

[13] Hampl SE Hassink SG Skinner AC Armstrong SC Barlow SE Bolling CF. Clinical practice guideline for the evaluation and treatment of children and adolescents with obesity. Pediatrics. (2023) 151:e2022060640. doi: 10.1542/9781610027052 36622115

[14] Gao X Fan JG Study Group of Liver and Metabolism, Chinese Society of Endocrinology. Diagnosis and management of non-alcoholic fatty liver disease and related metabolic disorders: consensus statement from the Study Group of Liver and Metabolism, Chinese Society of Endocrinology. J Diabetes. (2013) 5:406–15. doi: 10.1111/jdb.2013.5.issue-4 PMC3933762 23560695

[15] World Obesity Federation. Available online at: https://www.worldobesityday.org/assets/downloads/World_Obesity_Atlas_2023_Report.pdf (Accessed March 2023).

[16] Kim A Shah AS Nakamura T. Extracellular vesicles: A potential novel regulator of obesity and its associated complications. Children (Basel). (2018) 5:152. doi: 10.3390/children5110152 30445758 PMC6262587

[17] Lister NB Baur LA Felix JF Hill AJ Marcus C Reinehr T. Child and adolescent obesity. Nat Rev Dis Primers. (2023) 9:24. doi: 10.1038/s41572-023-00435-4 37202378

[18] Oses M Medrano M Margareto Sanchez J Portillo MP Aguilera CM Altmäe S. Peripheral blood mononuclear cells-expressed miRNA profiles derived from children with metabolic-associated fatty liver disease and insulin resistance. Pediatr Obes. (2022) 17:e12966. doi: 10.1111/ijpo.v17.12 36054529 PMC9787576

[19] Bianchi VE Locatelli V. Testosterone a key factor in gender related metabolic syndrome. Obes Rev. (2018) 19:557–75. doi: 10.1111/obr.12633 29356299

[20] Gilani A Stoll L Homan EA Lo JC. Adipose signals regulating distal organ health and disease. Diabetes. (2024) 73:169–77. doi: 10.2337/dbi23-0005 PMC10796297 38241508

[21] Hildreth AD Padilla ET Gupta M Wong YY Sun R Legala AR. Adipose cDC1s contribute to obesity-associated inflammation through STING-dependent IL-12 production. Nat Metab. (2023) 5:2237–52. doi: 10.1038/s42255-023-00934-4 PMC13020765 37996702

[22] Jebeile H Kelly AS O'Malley G Baur LA. Obesity in children and adolescents: epidemiology, causes, assessment, and management. Lancet Diabetes Endocrinol. (2022) 10:351–65. doi: 10.1016/S2213-8587(22)00047-X PMC9831747 35248172

[23] Ali A Al-Ani O Al-Ani F. Children's behaviour and childhood obesity. Pediatr Endocrinol Diabetes Metab. (2024) 30:148–58. doi: 10.5114/pedm.2024.142586 PMC11538919 39451187

[24] Wang Y Luo PL Bai H Fang YF Shen L Zhang MY. Correlation between serum uric,sex hormones and non-alcoholic fatty liver disease in obese children. Anhui Med Pharm J. (2024) 28:2205–12. doi: 10.3969/j.issn.1009-6469.2024.11.019

[25] Anderson EL Howe LD Jones HE Higgins JP Lawlor DA Fraser A. The prevalence of non-alcoholic fatty liver disease in children and adolescents: A systematic review and meta-analysis. PLoS One. (2015) 10:e0140908. doi: 10.1371/journal.pone.0140908 26512983 PMC4626023

[26] Marino L Jornayvaz FR. Endocrine causes of nonalcoholic fatty liver disease. World J Gastroenterol. (2015) 21:11053–76. doi: 10.3748/wjg.v21.i39.11053 PMC4607905 26494962

[27] Barbieri E Santoro N Umano GR. Clinical features and metabolic complications for non-alcoholic fatty liver disease (NAFLD) in youth with obesity. Front Endocrinol (Lausanne). (2023) 14:1062341. doi: 10.3389/fendo.2023.1062341 36733529 PMC9887046

[28] Kelly DM Jones TH. Testosterone: a metabolic hormone in health and disease. J Endocrinol. (2013) 217:R25–45. doi: 10.1530/JOE-12-0455 23378050

[29] Yang LJ Zhou JZ Zheng YF Hu X He ZY Du LJ. Association of non-alcoholic fatty liver disease with total testosterone in non-overweight/obese men with type 2 diabetes mellitus. J Endocrinol Invest. (2023) 46:1565–72. doi: 10.1007/s40618-023-02006-6 36725809

[30] Vosberg DE Parker N Shin J Pausova Z Paus T. The genetics of testosterone contributes to "femaleness/maleness" of cardiometabolic traits and type 2 diabetes. Int J Obes (Lond). (2022) 46:235–7. doi: 10.1038/s41366-021-00960-w 34480103

[31] Liao Z Vosberg DE Pausova Z Paus T. A shifting relationship between sex hormone-binding globulin and total testosterone across puberty in boys. J Clin Endocrinol Metab. (2022) 107:e4187–96. doi: 10.1210/clinem/dgac484 PMC9516180 35965384

[32] Song MJ Choi JY. Androgen dysfunction in non-alcoholic fatty liver disease: Role of sex hormone binding globulin. Front Endocrinol (Lausanne). (2022) 13:1053709. doi: 10.3389/fendo.2022.1053709 36482993 PMC9722756

[33] Sarkar M VanWagner LB Terry JG Carr JJ Rinella M Schreiner PJ. Sex hormone-binding globulin levels in young men are associated with nonalcoholic fatty liver disease in midlife. Am J Gastroenterol. (2019) 114:758–63. doi: 10.14309/ajg.0000000000000138 PMC6599461 30730350

[34] Qu X Donnelly R. Sex hormone-binding globulin (SHBG) as an early biomarker and therapeutic target in polycystic ovary syndrome. Int J Mol Sci. (2020) 21:8191. doi: 10.3390/ijms21218191 33139661 PMC7663738

[35] Urbano F Chiarito M Lattanzio C Messa A Ferrante M Francavilla M. Sex hormone-binding globulin (SHBG) reduction: the alarm bell for the risk of non-alcoholic fatty liver disease in adolescents with polycystic ovary syndrome. Children (Basel). (2022) 9:1748. doi: 10.3390/children9111748 36421197 PMC9689249

[36] Dong J Liu C Lu J Wang L Xie S Ji L. The relationship between sex hormone-binding protein and non-alcoholic fatty liver disease using Mendelian randomisation. Eur J Clin Invest. (2024) 54:e14082. doi: 10.1111/eci.14082 37605959

[37] Nobili V Alisi A Valenti L Miele L Feldstein AE Alkhouri N. NAFLD in children: new genes, new diagnostic modalities and new drugs. Nat Rev Gastroenterol Hepatol. (2019) 16:517–30. doi: 10.1038/s41575-019-0169-z 31278377

